# Racial/ethnic differences in the associations between trust in the U.S. healthcare system and willingness to test for and vaccinate against COVID-19

**DOI:** 10.1186/s12889-024-18526-6

**Published:** 2024-04-19

**Authors:** Judy Nanaw, Juliana S. Sherchan, Jessica R. Fernandez, Paula D. Strassle, Wizdom Powell, Allana T. Forde

**Affiliations:** 1 Division of Intramural Research, National Institute on Minority Health and Health Disparities, National Institutes of Health, Bethesda, MD USA; 2 Headspace Health, Durham, NC USA

**Keywords:** COVID-19 testing, COVID-19 vaccination, Disparities, Mistrust, Race/ethnicity

## Abstract

**Background:**

Trust in the healthcare system may impact adherence to recommended healthcare practices, including willingness to test for and vaccinate against COVID-19. This study examined racial/ethnic differences in the associations between trust in the U.S. healthcare system and willingness to test for and vaccinate against COVID-19 during the first year of the pandemic.

**Methods:**

This cross-sectional study used data from the REACH-US study, a nationally representative online survey conducted among a diverse sample of U.S. adults from January 26, 2021-March 3, 2021 (*N* = 5,121). Multivariable logistic regression estimated the associations between trust in the U.S. healthcare system (measured as “Always”, “Most of the time”, “Sometimes/Almost Never”, and “Never”) and willingness to test for COVID-19, and willingness to receive the COVID-19 vaccine. Racial/ethnic differences in these associations were examined using interaction terms and multigroup analyses.

**Results:**

Always trusting the U.S. healthcare system was highest among Hispanic/Latino Spanish Language Preference (24.9%) and Asian (16.7%) adults and lowest among Multiracial (8.7%) and Black/African American (10.7%) adults. Always trusting the U.S. healthcare system, compared to never, was associated with greater willingness to test for COVID-19 (AOR: 3.20, 95% CI: 2.38–4.30) and greater willingness to receive the COVID-19 vaccine (AOR: 2.68, 95% CI: 1.97–3.65).

**Conclusions:**

Trust in the U.S. healthcare system was associated with greater willingness to test for COVID-19 and receive the COVID-19 vaccine, however, trust in the U.S. healthcare system was lower among most marginalized racial/ethnic groups. Efforts to establish a more equitable healthcare system that increases trust may encourage COVID-19 preventive behaviors.

**Supplementary Information:**

The online version contains supplementary material available at 10.1186/s12889-024-18526-6.

## Background

Early in the SARS-CoV-2 (i.e., COVID-19) pandemic (March 2020-November 2020), there were vast racial/ethnic disparities in COVID-19 infection and mortality [1]. Racial/ethnic disparities in access and utilization of COVID-19 testing and vaccination were associated with differences in COVID-19 infection and mortality among Black, Latino, and American Indian/Alaska Native populations [2–5].

Barriers to COVID-19 testing and vaccination disparities in the first year of the COVID-19 pandemic included both structural (i.e., disparities in access and distribution to COVID-19 testing and vaccination sites) and attitudinal (i.e., beliefs/perceptions that reduced individuals’ willingness to seek out COVID-19 testing and vaccination) barriers [6–8]. Trust in the healthcare system was identified as a particularly important attitudinal barrier of racial/ethnic disparities in COVID-19 testing and vaccination [8, 9]. Both COVID-19 testing and vaccination required engagement with the healthcare system (i.e., COVID-19 testing was only available at health clinics prior to April 2021 [10]), and trust in the healthcare system was associated with COVID-19 testing and vaccination [11–15].

There is longstanding evidence that trust in the healthcare system varies between racial/ethnic groups. Prior to the pandemic, medical mistrust and distrust in the healthcare system (e.g., a cautious attitude towards the healthcare system or believing the healthcare system to be untrustworthy [16]) were more prevalent among marginalized racial/ethnic groups (e.g., medical mistrust and physician distrust were higher among Non-Hispanic Black and Hispanic adults compared to Non-Hispanic White adults [17, 18]). Non-Latino Black adults also reported higher general medical mistrust compared to Asian, Non-Latino White, and White-Latino adults [17–19]. These instances of higher medical mistrust and distrust in the healthcare system reflect historical and ongoing injustices experienced by marginalized racial/ethnic groups, including systemic racism and discrimination [13, 17, 19, 20].

Researchers suggest that trust in the healthcare system may partially explain racial/ethnic disparities in COVID-19 testing and vaccination rates [21, 22]. However, the literature largely focuses on COVID-19 vaccination [23]. Trust in the U.S. healthcare system, its association with COVID-19 testing, and whether associations differ across racial/ethnic groups remain underexplored. COVID-19 testing and vaccination require different degrees of invasiveness and engagement with the healthcare system, and trust in the healthcare system may differentially impact these practices. Further, invasive procedures may raise concerns related to historical medical mistreatment that may uniquely influence COVID-19 vaccination behaviors [24]. Yet to our knowledge, no studies examined COVID-19 testing and vaccination in the same study.

The present study addressed these gaps by examining the association between trust in the U.S. healthcare system and willingness to test for COVID-19 and receive the COVID-19 vaccine, and whether these associations varied across racial/ethnic groups in a large, nationally representative study.

## Methods

### Data source and study population

The Race-Related Experiences Associated with COVID-19 and Health in the United States (REACH-US) study is an online, nationally representative survey of U.S. adults that was conducted between January 26, 2021 and March 3, 2021. After informed consent was obtained from participants, the survey was administered by the nonpartisan YouGov, Inc. research firm, which uses an existing opt-in participant panel to conduct nationally representative surveys. Panel members were recruited and received panel rewards/incentives for their participation.

The REACH-US study included 5,500 adults from seven racial/ethnic subgroups (i.e., 500 American Indian/Alaska Native, 1000 Asian, 1000 Black/African American, 1000 Hispanic/Latino, 500 Multiracial, 500 Native Hawaiian/Pacific Islander, and 1000 White adults) living in the U.S. YouGov panel members were proximity matched to a target sample of U.S. adults generated from the 2018 American Community Survey 1-year data. Additional details on the recruitment methods, the stratified sampling approach to include a diverse set of racial/ethnic groups, the matching process, and propensity scoring to generate sample weights and nationally representative estimates within racial/ethnic groups are previously published [25].

Participants who already received at least one dose of the COVID-19 vaccine (*n* = 419) as well as those who did not provide responses for the trust item and/or the sociodemographic covariates were excluded from the present study analysis. The final sample size included 5,121 (weighted) participants (5,054 unweighted) (Figure S1). De-identified data were provided to the research team and the study was considered exempt, non-human subjects research by the Institutional Review Board of the National Institutes of Health.

### Measures

The study outcomes, willingness to test for COVID-19 and receive the COVID-19 vaccine, were captured by two separate items. At the time of data collection (January-March 2021), COVID-19 tests were largely available whereas COVID-19 vaccines were not yet available to all U.S. adults [26, 27]. Therefore, the items asked participants whether they had or would plan to get tested (if they developed symptoms) and whether they had or planned to receive the COVID-19 vaccine (once it was available to them). To capture willingness to receive the COVID-19 vaccine, participants who had already received at least one dose of the COVID-19 vaccine were not included in the analysis. Behavioral intentions were conceptualized as willingness to test for COVID-19 or receive the COVID-19 vaccine as described below.

#### Willingness to test for COVID-19

Participants were asked “Did you get tested for COVID-19?” Response options included: (1) Yes, I tested positive, (2) Yes, I tested negative, (3) Yes, I don’t know the results, (4) No, but I plan on getting tested soon, (5) No, but I would get tested in the future if I develop symptoms or come into contact with someone who has tested positive for COVID-19, and (6) No, and I don’t plan on getting tested now or in the future. Willingness to test for COVID-19 was dichotomized into whether participants had been tested or were willing to test for COVID-19 (responses 1 to 5 coded as 0) versus unwilling to test for COVID-19 (response 6 coded as 1).

#### Willingness to receive the COVID-19 vaccine

Participants were asked “Do you plan to get the COVID-19 vaccine once it becomes available?” Response options included (1) Definitely not, (2) Probably not, (3) Probably yes, (4) Definitely yes, (5) I have received one dose of the COVID-19 vaccine, and (6) I have received two doses of the COVID-19 vaccine. Willingness to receive the COVID-19 vaccine was treated as an ordinal variable: (1) “Definitely not”, (2) Probably not”, (3) “Probably yes”, and (4) “Definitely yes”. Given that willingness to receive the COVID-19 vaccine was captured as plans to receive the COVID-19 vaccine when it became available, participants who had already received at least one dose of the COVID-19 vaccine were excluded.

#### Trust in the U.S. healthcare system

Participants were asked “How often do you trust the healthcare system (e.g., doctors, nurses)?” Response options included: (1) Always, (2) Most of the time, (3) Sometimes, (4) Almost never, (5) Never. Consistent with previous literature [28], these responses were categorized as “Always” (coded as 3), “Most of the time” (coded as 2), “Sometimes/Almost Never” (coded as 1), and “Never” (coded as 0).

#### Race/ethnicity and sociodemographic covariates

Racial/ethnic group membership was self-identified by participants and included American Indian/Alaska Native, Asian, Black/African American, Hispanic/Latino, Multiracial, Native Hawaiian/Pacific Islander, and White. Hispanic/Latino participants were further stratified into English Language Preference (ELP) and Spanish Language Preference (SLP) depending on their survey language.

Other sociodemographic characteristics included age (i.e., 18–34, 35–49, 50–64, 65 and older), gender (i.e., man, woman, nonbinary, transgender, not listed), educational attainment (i.e., high school or less, some college or 2-year college, 4-year college, post-graduate), and annual household income (i.e., <$20,000, $20,000–49,999, $50,000–99,999, ≥$100,000). Sociodemographic characteristics previously associated with COVID-19 testing and vaccination included health insurance coverage (i.e., not covered, covered), political ideology (i.e., conservative, liberal, moderate, not sure), and high-risk chronic health condition (i.e., not high-risk, high-risk).

### Statistical analyses

Descriptive analyses were used to assess the distribution of sociodemographic characteristics and trust in the U.S. healthcare system, overall and stratified by race/ethnicity, as well as willingness to test for and vaccinate against COVID-19, overall and stratified by trust in the U.S. healthcare system.

In the overall models using adjusted logistic regression, trust in the U.S. healthcare system was regressed on willingness to test for and willingness to receive the COVID-19 vaccine (in two separate models) to produce adjusted odds ratio estimates (AORs). Adjusted models included race/ethnicity, age, gender, annual household income, educational attainment, health insurance, political ideology, and high-risk chronic health condition.

Racial/ethnic differences in the associations between trust in the U.S. healthcare system and willingness to test for and vaccinate against COVID-19 were each assessed using an interaction model and multigroup analyses (see conceptual model, Figure S1). An interaction term for racial/ethnic group and trust in the U.S. healthcare system was added to the fully adjusted logistic regression models for willingness to test for and vaccinate against COVID-19. Multigroup analyses were used to further examine the interaction models by generating estimates of the associations between trust in the U.S. healthcare system and each of the outcomes within each racial/ethnic group, adjusting for the covariates across racial/ethnic groups.

Regression analyses were conducted using R Version 4.1.2 and multigroup analyses were conducted using M*plus* Version 8.6 [29]. All analyses were weighted to be nationally representative within each racial/ethnic group.

## Results

Sociodemographic characteristics of the study population varied across racial/ethnic groups (Table 1). Overall, 14.6% of participants reported always trusting the U.S. healthcare system and 10.8% reported never trusting the U.S. healthcare system (Table 1); however, trust significantly varied across racial/ethnic groups. The prevalence of always trusting the U.S. healthcare system was highest among Hispanic/Latino SLP (24.9%) and Asian (16.7%) adults and lowest among Multiracial (8.7%) and Black/African American adults (10.7%) (Table 1). The prevalence of never trusting the U.S. healthcare system was highest among Native Hawaiian/Pacific Islander (17.7%) and Hispanic/Latino ELP adults (13.5%) and lowest among Multiracial (5.5%) and White adults (8.2%).

**Table 1:** Study population characteristics across racial/ethnic groups (weighted)

	Total (*N* = 5,121)	AI/AN (*n* = 456)	Asian (*n* = 923)	Black/AA (*n* = 946)	Hispanic/ Latino ELP (*n* = 462)	Hispanic/ Latino SLP (*n* = 487)	Multiracial (*n* = 471)	NH/PI (*n* = 460)	White (*n* = 916)
Age, %									
18–34	35.7	35.3	34.4	34.5	44.0	36.3	48.7	37.4	26.2
35–49	27.2	24.1	29.7	24.1	27.1	36.4	24.9	34.3	22.0
50–64	23.9	26.0	23.3	27.1	13.5	24.2	17.9	22.2	29.4
≥65	13.2	14.6	12.5	14.3	15.5	3.0	8.5	6.1	22.5
Gender, %									
Man	44.3	35.7	45.7	46.6	49.8	38.7	46.7	33.6	48.9
Woman	53.9	61.5	52.8	52.7	49.1	60.9	46.3	63.6	50.1
Nonbinary	1.2	2.5	0.7	0.6	0.6	0.2	4.8	0.3	0.9
Transgender	0.3	0.3	0.2	0.1	0.1	0.2	0.3	2.0	0.0
Not listed	0.4	0.0	0.6	0.0	0.3	0.0	1.9	0.5	0.1
Education, %									
High school or less	40.6	43.4	25.0	42.7	53.0	66.9	33.0	46.9	33.5
Some college, 2-year college	33.4	45.2	24.6	38.2	32.0	21.1	37.7	38.2	34.0
4-year college	16.2	7.1	29.8	11.7	8.3	9.3	19.4	11.4	20.3
Post-graduate	9.7	4.2	20.6	7.5	6.7	2.7	9.8	3.4	12.2
Annual Household Income, %									
< $20,000	30.4	42.9	18.7	41.9	30.6	32.3	25.1	38.8	21.2
$20,000–49,000	31.5	31.5	26.6	30.6	35.4	48.2	28.3	27.3	30.0
$50,000-100,000	24.5	18.5	28.6	18.8	24.0	15.5	32.1	25.4	29.9
*≥* $100,000	13.7	7.0	26.1	8.7	10.0	4.1	14.5	8.6	18.9
Political Ideology, %									
Conservative	23.7	29.1	18.8	15.5	22.0	17.9	16.3	26.3	41.0
Liberal	29.3	21.8	33.1	31.3	32.8	26.5	41.9	19.2	25.4
Moderate	32.4	30.0	38.7	36.8	32.1	28.0	31.5	30.2	26.6
Not sure	14.6	19.0	9.4	16.4	13.2	27.6	10.3	24.3	7.1
Health Insurance Coverage, %									
Covered	84.0	87.2	90.4	83.8	81.6	57.7	88.0	82.8	90.1
Not Covered	16.0	12.8	9.6	16.2	18.4	42.3	12.0	17.2	9.9
High-Risk Chronic Health Condition, %									
High-risk	40.7	50.3	32.6	47.4	36.0	29.7	40.6	41.6	45.3
Not high-risk	59.3	49.7	67.4	52.6	64.0	70.3	59.4	58.4	54.7
Willingness to Test for COVID-19, %									
Unwilling to test	17.1	20.4	16.8	17.9	14.7	9.8	18.1	13.9	21.0
Willing to test	82.9	79.6	83.2	82.1	85.3	90.2	81.9	86.1	79.0
Willingness to Receive the COVID-19 Vaccine, %									
Definitely not	17.5	29.1	8.6	22.1	13.9	11.2	18.5	17.0	20.8
Probably not	17.6	15.8	12.9	22.9	16.5	12.3	18.9	27.6	15.6
Probably yes	28.9	27.6	31.5	28.5	33.7	35.9	23.8	29.0	23.9
Definitely yes	36.0	27.6	47.1	26.5	36.0	40.5	38.9	26.4	39.6
Trust in the U.S. healthcare system, %									
Never	10.8	9.2	10.4	11.9	13.5	12.1	5.5	17.7	8.2
Almost Never/Sometimes	37.6	42.5	29.9	50.0	36.0	34.4	43.5	40.6	28.4
Most of the time	37.0	33.1	43.0	27.5	35.6	28.6	42.2	29.0	49.1
Always	14.6	15.2	16.7	10.7	14.9	24.9	8.7	12.8	14.4

^a^ Weighted to be nationally representative within each racial/ethnic group

^b^ AI/AN = American Indian/Alaska Native; Black/AA = Black/African American; Hispanic/Latino ELP = Hispanic/Latino English Language Preference; Hispanic/Latino SLP = Hispanic/Latino Spanish Language Preference; NH/PI = Native Hawaiian/Pacific Islander

^c^ The high-risk chronic health condition covariate was created based on the February 2021 Centers for Disease Control (CDC) list of

medical conditions that increase risk of severe illness due to COVID-19 (Kates, J., L. Dawson, and J. Tolbert. *The Next Phase of*

*Vaccine Distribution: High-Risk Medical Conditions*. 2021; Available from: https://www.kff.org/policy-watch/the-next-phase-of-vaccine-distribution-high-risk-medical-conditions/). High-risk chronic health condition was coded as 1 if participants reported at least one medical condition that was considered high-risk according to the CDC.

^d^ All categories of sociodemographic characteristics varied significantly between racial/ethnic group (*p* < 0.05)

Most participants (82.9%) were willing to test for COVID-19 and 36.0% of participants reported that they would definitely receive the COVID-19 vaccine (Table 1), but both willingness to test for and receive the COVID-19 vaccine varied by levels of trust (Figures S3-S4). After adjustment, compared to those who never trusted the U.S. healthcare system, participants who trusted the U.S. healthcare system “Always” (AOR: 3.20, 95% CI: 2.38–4.30), “Most of the time” (AOR: 3.41, 95% CI: 2.69–4.33) and “Sometimes/Almost Never” (AOR: 2.06, 95% CI: 1.65–2.57) were more willing to test for COVID-19 (Table 2; unadjusted ORs in Table S1).

**Table 2:** Trust in the U.S. healthcare system and willingness to test for COVID-19 and receive the COVID-19 vaccine

	Willingness to test for COVID-19	Willingness to receive the COVID-19 vaccine
	AOR [95% CI]	AOR [95% CI]
**Trust in the U.S. healthcare system**		
Always	3.20 [2.38–4.30]	2.68 [1.97–3.65]
Most of the time	3.41 [2.69–4.33]	2.71 [2.12–3.46]
Sometimes/Almost Never	2.06 [1.65–2.57]	1.65 [1.32–2.07]

^a^ Weighted to be nationally representative within each racial/ethnic group

^b^ AOR = Adjusted Odds Ratio. Adjusted for race/ethnicity, age, gender, annual household income, education level, political ideology, health insurance coverage, and high-risk chronic health condition

^c^ Reference group = Never trusting the U.S. healthcare system

Similarly, those who trusted the U.S. healthcare system “Always” (AOR: 2.68, 95% CI: 1.97–3.65), “Most of the time” (AOR: 2.71, 95% CI: 2.12–3.46), and “Sometimes/Almost Never” (AOR: 1.65, 95% CI: 1.32–2.07) were more willing to receive the COVID-19 vaccine, compared to those who never trusted the U.S. healthcare system.

The direction of the associations between trust in the U.S. healthcare system and willingness to test for COVID-19 remained consistent across racial/ethnic groups (i.e., those who reported higher levels of trust versus “Never” trusting the U.S. healthcare system were more willing to test for COVID-19), however the magnitude of associations varied across racial/ethnic groups (global significance test in overall interaction model < 0.01) (see Table S2 for interactions between trust in the U.S. healthcare system and racial/ethnic group). The AORs for “Always” trusting the U.S. healthcare system (versus “Never”) and willingness to test for COVID-19 were particularly high among Hispanic/Latino SLP (AOR: 16.28, 95% CI: 6.24–42.47) and Hispanic/Latino ELP adults (AOR: 12.25, 95% CI: 4.05–37.07), compared to the other racial/ethnic groups (AORs: 1.04–3.10) (Table 3; unadjusted ORs in Table S3).

**Table 3:** Trust in the U.S. healthcare system and willingness to test for COVID-19 across racial/ethnic groups (multigroup analysis)

	Willingness to test for COVID-19							
	AI/AN	Asian	Black/AA	Hispanic/ Latino ELP	Hispanic/ Latino SLP	Multiracial	NH/PI	White
**Trust in the U.S. healthcare system**								
Always	1.29 [0.49–3.39]	3.10 [1.59–6.06]	2.46 [1.24–4.88]	12.25 [4.05–37.07]	16.28 [6.24–42.47]	1.04 [0.47–2.28]	2.47 [0.67–9.13]	3.07 [1.56–6.02]
Most of the time	3.28 [1.79–5.99]	3.06 [1.94–4.84]	2.49 [1.56–3.96]	2.99 [1.62–5.52]	7.46 [3.64–15.30]	2.78 [1.54–5.03]	5.01 [1.98–12.67]	2.31 [1.53–3.51]
Sometimes/Almost Never	1.70 [0.97–2.96]	1.40 [0.81–2.40]	2.30 [1.56–3.38]	2.53 [1.47–4.34]	3.59 [2.00–6.42]	1.44 [0.88–2.35]	2.73 [1.46–5.08]	1.35 [0.88–2.06]

^a^ Weighted to be nationally representative within each racial/ethnic group

^b^ AOR = Adjusted Odds Ratio. Adjusted for age, gender, annual household income, education level, political ideology, health insurance coverage, and high-risk chronic health condition

^c^ Reference group = Never trusting the U.S. healthcare system

^d^ AI/AN = American Indian/Alaska Native; Black/AA = Black/African American; Hispanic/Latino ELP = Hispanic/Latino English Language Preference; Hispanic/Latino SLP = Hispanic/Latino Spanish Language Preference; NH/PI = Native Hawaiian/Pacific Islander

The AORs for trusting the U.S. healthcare system “Most of the time” versus “Never” were also high among Hispanic/Latino SLP (AOR: 7.46, 95% CI: 3.64–15.30) and Native Hawaiian/Pacific Islander adults (AOR: 5.01, 95% CI: 1.98–12.67). There was some variation in the confidence intervals estimated across racial/ethnic groups, with wide confidence intervals observed for some of the estimated AORs (Table 3).

The associations between trust in the U.S. healthcare system and willingness to receive the COVID-19 vaccine also varied across racial/ethnic groups (global significance test in overall interaction model < 0.01) (see Table S4 for interactions between trust in the U.S. healthcare system and racial/ethnic group). Similarly, the AORs for “Always” trusting the U.S. healthcare system (versus “Never”) and willingness to receive the COVID-19 vaccine were high among Hispanic/Latino SLP (AOR: 5.63, 95% CI: 3.39–9.36) and Hispanic/Latino ELP adults (AOR: 3.89, 95% CI: 2.13–7.13), compared to the other racial/ethnic groups (AORs: 1.33–2.21) (Table 4; unadjusted ORs in Table S5). However, the associations between trust in the U.S. healthcare system and willingness to receive the COVID-19 vaccine were less consistent across racial/ethnic groups compared to willingness to test for COVID-19 (i.e., wide confidence intervals for many estimated AORs).

**Table 4:** Trust in the U.S. healthcare system and willingness to receive the COVID-19 vaccine across racial/ethnic groups (multigroup analysis)

	Willingness to receive the COVID-19 vaccine							
	AI/AN	Asian	Black/AA	Hispanic/ Latino ELP	Hispanic/ Latino SLP	Multiracial	NH/PI	White
**Trust in the U.S. healthcare system**								
Always	1.77 [0.83–3.81]	1.79 [1.05–3.05]	1.42 [0.86–2.35]	3.89 [2.13–7.13]	5.63 [3.39–9.36]	1.33 [0.61–2.86]	1.63 [0.79–3.40]	2.21 [1.36–3.59]
Most of the time	1.37 [0.86–2.18]	2.00 [1.45–2.77]	0.96 [0.68–1.35]	1.49 [1.02–2.17]	2.43 [1.61–3.67]	1.42 [0.98–2.05]	1.33 [0.85–2.08]	1.46 [1.06–2.02]
Sometimes/Almost Never	0.58 [0.38–0.89]	1.28 [0.84–1.94]	0.71 [0.53–0.95]	1.18 [0.82–1.68]	1.93 [1.29–2.87]	0.73 [0.51–1.04]	0.90 [0.61–1.33]	0.79 [0.56–1.11]

^a^ Weighted to be nationally representative within each racial/ethnic group

^b^ AOR = Adjusted Odds Ratio. Adjusted for age, gender, annual household income, education level, political ideology, health insurance coverage, and high-risk chronic health condition

^c^ Reference group = Never trusting the U.S. healthcare system

^d^ AI/AN = American Indian/Alaska Native; Black/AA = Black/African American; Hispanic/Latino ELP = Hispanic/Latino English Language Preference; Hispanic/Latino SLP = Hispanic/Latino Spanish Language Preference; NH/PI = Native Hawaiian/Pacific Islander

## Discussion

Trust in the U.S. healthcare system was associated with greater willingness to test for and receive the COVID-19 vaccine in the overall study population. Those who trusted the U.S. healthcare system were two to three times as likely to be willing to test for COVID-19 and up to two times as likely to be willing to receive the COVID-19 vaccine. The associations between trust in the U.S. healthcare system and willingness to test for and receive the COVID-19 vaccine were consistent when assessing different levels of trust (i.e., comparing always, most of time, sometimes/almost never versus never trusting the U.S. healthcare system). These findings generally reflect prior literature on trust in the U.S. healthcare system and other preventive health behaviors (e.g., breast, cervical, and prostate cancer screening services [30, 31]).

Multigroup analysis revealed two important racial/ethnic differences in the associations between trust in the U.S. healthcare system and willingness to test for and receive the COVID-19 vaccine. The associations between trust in the U.S. healthcare system and willingness to test for and receive the COVID-19 vaccine were high among Hispanic/Latino adults. This is consistent with previous literature that found higher levels of trust in medical providers were significantly associated with higher levels of healthcare utilization among Hispanic/Latino adults [32]. The authors suggested that Latino cultural values may facilitate relationship building and influence how Hispanic/Latino adults interact with the healthcare system. In addition, a recent study among Hispanic Americans found most (56%) reported positive ratings for the quality of their recent healthcare and most (51%) thought health outcomes for Hispanic people have improved in the past 20 years [33]. These positive attitudes toward the U.S. healthcare system may partially explain the strong relationship between trust in the U.S. healthcare system and willingness to test for and receive the COVID-19 vaccine among Hispanic/Latino adults.

A second important finding from the multigroup analysis revealed that for American Indian/Alaska Native and Black/African American adults, trust in the U.S. healthcare system did not impact willingness to receive the COVID-19 vaccine. Among the remaining racial/ethnic groups in the present study, participants were more willing to receive the COVID-19 vaccine when they had higher levels of trust (always or most of the time) in the U.S. healthcare system. These findings may be attributed to historical injustices by the U.S. government and healthcare system toward American Indian/Alaska Native and Black/African American adults [34–36] that may complicate the relationship between trust in the healthcare system and preventive health behaviors.

Several limitations should be noted. The study population was matched and weighted to obtain a nationally representative sample, yet selection bias may still exist. The survey was conducted online, which may have created technology barriers for low-income individuals and individuals that live in rural places with broadband connectivity issues. The survey was also only conducted in English and Spanish (Hispanic/Latino only). Given that limited English proficiency has been associated with lower education and income [37, 38], excluding adults that do not speak English may have resulted in a sample population with a higher level of education, higher income, and better access to healthcare. Given that less education has been associated with greater healthcare distrust and COVID-19 vaccine hesitancy, the associations between trust in the U.S. healthcare system and willingness to test for and receive the COVID-19 vaccine may have been stronger in the present study sample than in the full U.S. population. This limitation may have especially impacted results for Asian participants given that an estimated 31.9% of Asian adults in the U.S. have limited English proficiency [39]. Additionally, the study was cross-sectional which limited the ability to make causal inferences as well as examine possible changes in behavioral intentions throughout the pandemic. For example, this survey was administered shortly after the Pfizer and Moderna COVID-19 vaccines were approved for emergency use authorization. Willingness to vaccinate may have increased over the study period as more people were allowed to receive the vaccine, people saw others get vaccinated, or vaccination became mandated. Moreover, trust in the U.S. healthcare system may not have been fully captured as it was measured using a single survey question. Future studies could consider additional measures of trust (e.g., trust in healthcare providers and specific COVID-19 health services).

Furthermore, because inferring causality was not feasible, potential barriers may impede the determination of causal relationships. Amidst the societal and political conflicts arising from divergent perspectives on COVID-19, COVID-19 testing, and COVID-19 vaccination, discerning whether trust in the U.S. healthcare system, as measured in the REACH-US study (“How often do you trust the healthcare system (e.g., doctors, nurses? ),” was cultivated or diminished before or after the pandemic proves challenging. In essence, determining whether respondents began distrusting the healthcare system due to the pandemic introduces the potential obstacle of reverse causation.

Despite the complexities in causal inference, it is crucial to report associations for several reasons. Associations between trust in the U.S. healthcare system and willingness to test for and vaccinate against COVID-19 offer valuable insights that can inform decision-making across various domains, including healthcare, education, and public policy. Moreover, these associations can serve as a foundation for formulating hypotheses that can be tested to explore potential causal relationships between the variables. Furthermore, the identified associations in our study can pinpoint areas warranting deeper investigation, guiding researchers to study our specific variables more closely to establish causation. Given the significance of the COVID-19 topic and its potential implications for racial/ethnic inequities, reporting cross-sectional associations contribute to public awareness and communicates findings to a wide audience.

Despite these limitations, this study had several strengths. This study included a nationally representative study population of racially/ethnically diverse participants in the U.S. In addition, this study explored both the association between trust in the U.S. healthcare system and COVID-19 testing, as well as COVID-19 vaccination which allowed for assessing trends for two types of COVID-19 preventive behaviors. Trust in the U.S. healthcare system may play a more important role in COVID-19 testing behaviors compared to receiving the COVID-19 vaccine. These findings are valuable to mitigation efforts of the COVID-19 pandemic, since testing and vaccination are two COVID-19-related preventive behaviors that differ in the degree of engagement in the healthcare system and degree of invasiveness (i.e., a nasal swab/saliva test versus injections). Furthermore, this study contributes to a growing body of literature on trust in the U.S. healthcare system and COVID-19 preventive behaviors which has been mixed [19, 40]).

This study has important implications for COVID-19 health services disparities. Lower trust in the U.S. healthcare system among many marginalized racial/ethnic groups is likely attributed to systemic inequities related to COVID-19 outcomes, spread of misinformation, and changing guidelines by public health officials. Increasing trust in the U.S. healthcare system may be needed to encourage COVID-19 testing and vaccination among U.S. adults from marginalized racial/ethnic groups and may be especially impactful among Hispanic/Latino adults. In addition, the relationship between trust in the U.S. healthcare system and willingness to receive the COVID-19 vaccine among American Indian/Alaska Native and Black/African American adults may require further investigation. Future studies should examine whether increasing trust in the U.S. healthcare system increases the likelihood of testing for and vaccinating against COVID-19 over time, thus impacting the spread and health burden of COVID-19.

### Electronic supplementary material

Below is the link to the electronic supplementary material.

Supplementary Material 1

## Data Availability

The data used in the current study are available from the corresponding author on reasonable request.

## Declarations

### Disclaimers

The contents and views in this manuscript are those of the authors and should not be construed to represent the views of the National Institute on Minority Health and Health Disparities, National Institutes of Health, or the Department of Health and Human Services.

## References

[1] Wong MS (2021). Time Trends in Racial/Ethnic differences in COVID-19 infection and mortality. Int J Environ Res Public Health.

[2] Mody A (2021). Understanding drivers of Coronavirus Disease 2019 (COVID-19) racial disparities: a Population-Level analysis of COVID-19 testing among black and white populations. Clin Infect Diseases: Official Publication Infect Dis Soc Am.

[3] Cardona S (2021). Vaccination disparity: quantifying racial inequity in COVID-19 Vaccine Administration in Maryland. J Urb Health.

[4] Gehlbach D (2022). Perceptions of the coronavirus and COVID-19 testing and vaccination in Latinx and indigenous Mexican immigrant communities in the Eastern Coachella Valley. BMC Public Health.

[5] Arrazola J (2020). COVID-19 mortality among American Indian and Alaska native persons– 14 states, January-June 2020. MMWR Morb Mortal Wkly Rep.

[6] Lieberman-Cribbin W (2020). Disparities in COVID-19 testing and positivity in New York City. Am J Prev Med.

[7] DiRago NV (2022). COVID-19 vaccine rollouts and the Reproduction of urban spatial inequality: disparities within large US cities in March and April 2021 by Racial/Ethnic and socioeconomic composition. J Urb Health.

[8] Fisk RJ (2021). Barriers to vaccination for coronavirus disease 2019 (COVID-19) control: experience from the United States. Global Health J.

[9] Yuan H, et al. Different roles of interpersonal trust and institutional trust in COVID-19 pandemic control. Volume 293. Social Science & Medicine; 2022. p. 114677. 10.1016/j.socscimed.2021.114677 PMC8692240 35101260

[10] Rubin R (2021). COVID-19 testing moves out of the clinic and into the home. JAMA.

[11] Egelko A (2020). Do I have to be tested? Understanding reluctance to be screened for COVID-19. Am J Public Health.

[12] Kim SJ et al. Addressing Racial/Ethnic equity in Access to COVID-19 Testing through Drive-Thru and Walk-In Testing sites in Chicago. Med Res Arch, 2021. 9(5). 10.18103/mra.v9i5.2430 PMC8186439 34109272

[13] Bogart LM (2021). COVID-19 Related Medical Mistrust, Health impacts, and potential vaccine hesitancy among Black americans Living with HIV. J Acquir Immune Defic Syndr.

[14] Turhan Z, Dilcen HY, Dolu İ (2022). The mediating role of health literacy on the relationship between health care system distrust and vaccine hesitancy during COVID-19 pandemic. Curr Psychol.

[15] Allen JD (2022). Medical mistrust, discrimination, and COVID-19 vaccine behaviors among a national sample U.S. adults. SSM - Popul Health.

[16] Jennings W (2021). How trust, mistrust and distrust shape the governance of the COVID-19 crisis. J Eur Public Policy.

[17] Bazargan M, Cobb S, Assari S (2021). Discrimination and medical mistrust in a racially and ethnically diverse sample of California adults. Ann Fam Med.

[18] Armstrong K (2007). Racial/ethnic differences in physician distrust in the United States. Am J Public Health.

[19] Smith AC (2022). An investigation of associations between race, ethnicity, and past experiences of discrimination with medical mistrust and COVID-19 protective strategies. J Racial Ethnic Health Disparities.

[20] Alang S, McAlpine DD, Hardeman R (2020). Police brutality and mistrust in Medical Institutions. J Racial Ethn Health Disparities.

[21] Escobar GJ (2021). Racial disparities in COVID-19 testing and outcomes: Retrospective Cohort Study in an Integrated Health System. Ann Intern Med.

[22] Jacobson M (2021). Racial and ethnic disparities in SARS-CoV-2 testing and COVID-19 outcomes in a Medicaid Managed Care Cohort. Am J Prev Med.

[23] Thompson HS (2021). Factors Associated with Racial/Ethnic group–based Medical Mistrust and perspectives on COVID-19 Vaccine Trial Participation and Vaccine Uptake in the US. JAMA Netw Open.

[24] Nuriddin A, Mooney G, White AIR (2020). Reckoning with histories of medical racism and violence in the USA. Lancet.

[25] Fernandez JR (2023). County-level barriers in the COVID-19 vaccine coverage index and their associations with willingness to receive the COVID-19 vaccine across racial/ethnic groups in the US. Front Public Health.

[26] Risanger S (2021). Selecting pharmacies for COVID-19 testing to ensure access. Health Care Manag Sci.

[27] Hughes MM (2021). County-Level COVID-19 Vaccination Coverage and Social Vulnerability - United States, December 14, 2020-March 1, 2021. MMWR Morb Mortal Wkly Rep.

[28] Szilagyi PG (2021). The role of trust in the likelihood of receiving a COVID-19 vaccine: results from a national survey. Prev Med.

[29] Muthén LK, Muthén B (2017). Mplus user’s guide. Statistical analysis with latent variables. Eighth Edition.

[30] Yang T-C, Matthews SA, Hillemeier MM (2011). Effect of Health Care System Distrust on breast and cervical Cancer screening in Philadelphia, Pennsylvania. Am J Public Health.

[31] Yang T-C, Matthews SA, Anderson RT (2013). Prostate Cancer Screening and Health Care System Distrust in Philadelphia. J Aging Health.

[32] Boyas JF, Negi NJ, Valera P (2017). Factors Associated to Health Care Service use among latino day laborers. Am J Men’s Health.

[33] Funk C, Lopez MH. Hispanic Americans’ experiences with health care. 2022; https://www.pewresearch.org/science/2022/06/14/hispanic-americans-experiences-with-health-care/.

[34] Evans-Campbell T (2012). Indian Boarding School Experience, Substance Use, and Mental Health among Urban two-Spirit American Indian/Alaska natives. Am J Drug Alcohol Abus.

[35] Stewart TJ, Gonzalez VM (2023). Associations of historical trauma and racism with health care system distrust and mental health help-seeking propensity among American Indian and Alaska Native college students. Cult Divers Ethnic Minor Psychol.

[36] Wailoo K (2018). Historical aspects of race and medicine: the case of J. Marion Sims. JAMA.

[37] Gulati RK, Hur K (2022). Association between Limited English proficiency and Healthcare Access and utilization in California. J Immigr Minor Health.

[38] Kim J, Ford K-L, Kim G (2019). Geographic disparities in the relation between English proficiency and Health Insurance Status among older latino and Asian immigrants. J Cross-Cult Gerontol.

[39] AAPI Data. 2022; https://aapidata.com/wp-content/uploads/2022/06/State-AANHPIs-National-June2022.pdf.

[40] LaVeist TA, Isaac LA, Williams KP (2009). Mistrust of health care organizations is associated with underutilization of health services. Health Serv Res.

