# Supplementary material for: Racial/ethnic differences in the associations between trust in the U.S. healthcare system and willingness to test for and vaccinate against COVID-19
Source: BMC Public Health. 2024 Apr 19;24:1084. doi: 10.1186/s12889-024-18526-6 (PMC11027359; doi:10.1186/s12889-024-18526-6)
Supplement: Supplementary file 1 — Supplementary Material 1 [file 12889_2024_18526_MOESM1_ESM.docx]

Supplemental Material

Figure S1. Flow Chart of Participant Exclusions in the REACH-US Study (unweighted)

Total REACH-US study sample

N= 5,500

Received at least one dose of the COVID-19 vaccine

n = 419

Missing responses for the trust item and/or sociodemographic covariates

n = 27

Final unweighted sample

N = 5,054

Figure S2. Conceptual model for trust in the U.S. healthcare system and willingness to test for COVID-19 and receive the COVID-19 vaccine.

**Race–Ethnicity**

**Willingness to Test for COVID-19**

**Willingness to Receive the**

**COVID-19 Vaccine**

Figure S3. Willingness to test for COVID-19, stratified by trust in the U.S. healthcare system

^a^ Weighted to be nationally representative within each racial/ethnic group.

^b^ Unwilling to test for COVID-19 included: No, and I don’t plan on getting tested now or in the future.

^c^ Willing to test for COVID-19 included: Yes, I tested positive Yes, I tested negative, Yes, I don’t know the results, No, but I

plan on getting tested soon, No, but I would get tested in the future if I develop symptoms or come into contact with

someone who has tested positive for COVID-19.

^d^ Willingness to test for COVID-19 varied significantly between levels of trust (p<0.01).

Figure S4: Willingness to receive the COVID-19 vaccine, stratified by trust in the U.S. healthcare system

^a^ Weighted to be nationally representative within each racial/ethnic group.

^b^ Unwilling to receive the COVID-19 vaccine included: Definitely not, Probably not.

^c^ Willingness to receive the COVID-19 vaccine included: Definitely not, Probably not, Probably yes, Definitely yes.

^d^ Willingness to receive the COVID-19 vaccine varied significantly between levels of trust (p<0.01).

Table S1. Trust in the U.S. healthcare system and willingness to test for COVID-19 and receive the COVID-19 vaccine

|  | Willingness to test for COVID-19 | Willingness to receive the COVID-19 vaccine |
| --- | --- | --- |
|  | OR [95% CI] | OR [95% CI] |
| Trust in the U.S. healthcare system |  |  |
| Always | 3.69 [2.79 – 4.88] | 3.38 [2.53 – 4.51] |
| Most of the time | 3.77 [3.01 – 4.70] | 3.07 [2.45 – 3.85] |
| Sometimes/Almost Never | 2.31 [1.88 – 2.85] | 1.67 [1.35 – 2.06] |

^a^ Weighted to be nationally representative within each racial/ethnic group.

^b^ OR=Odds Ratio (unadjusted).

^c^ Reference group: Never trusting the U.S. healthcare system.

Table S2. Racial/ethnic differences in associations between trust in the U.S. healthcare system and willingness to test for COVID-19 using interaction terms

|  | Always | Most of the time | Sometimes/Almost never |
| --- | --- | --- | --- |
|  | AOR [95% CIs]  p-value | | |
| Interaction terms |  |  |  |
| Trust*AI/AN (versus White) | 0.24 [0.08 – 0.72]  0.01 | 0.80 [0.30 – 2.11]  0.46 | 0.71 [0.28 – 1.78]  0.46 |
| Trust *Asian (versus White) | 0.78 [0.30 – 2.04]  0.61 | 1.01 [0.47 – 2.15]  0.98 | 0.79 [0.37 – 1.68]  0.54 |
| Trust*Black/AA (versus White) | 0.52 [0.19 –1.39]  0.19 | 0.69 [0.32 – 1.48]  0.34 | 1.09 [0.53 – 2.25]  0.81 |
| Trust*H/L ELP (versus White) | 1.93 [0.40 – 9.25]  0.41 | 0.62 [0.25 – 1.53]  0.30 | 0.89 [0.36 – 2.17]  0.79 |
| Trust*H/L SLP (versus White) | 1.16 [0.29 – 4.73]  0.83 | 0.70 [0.24 – 2.07]  0.52 | 0.57 [0.21 – 1.52]  0.26 |
| Trust*Multiracial (versus White) | 0.26 [0.07 – 0.97]  0.04 | 0.94 [0.32 – 2.73]  0.90 | 0.83 [0.29 – 2.34]  0.73 |
| Trust*NH/PI (versus White) | 0.15 [0.05 – 0.49]  0.00 | 0.41 [0.15 – 1.11]  0.08 | 0.38 [0.16 – 0.93]  0.03 |

^a^ Weighted to be nationally representative within each racial/ethnic group.

^b^ AOR=Adjusted Odds Ratio.

^c^ AI/AN=American Indian/Alaska Native; Black/AA=Black/African American; H/L ELP=Hispanic/Latino English

Language Preference; H/L SLP=Hispanic/Latino Spanish Language Preference; NH/PI=Native

Hawaiian/Pacific Islander.

Table S3. Trust in the U.S. healthcare system and willingness to test for COVID-19 across racial/ethnic groups-(multigroup analysis)

|  | **Willingness to test for COVID-19** | | | | | | | |
| --- | --- | --- | --- | --- | --- | --- | --- | --- |
|  | **AI/AN** | **Asian** | **Black/AA** | **Hispanic/**  **Latino ELP** | **Hispanic/**  **Latino SLP** | **Multiracial** | **NH/PI** | **White** |
| **Trust in the U.S. healthcare system** |  |  |  |  |  |  |  |  |
| Always | 1.55  [0.61 – 3.94] | 3.97  [2.07 – 7.61] | 2.84  [1.46 – 5.51] | 14.25  [4.76 – 42.72] | 15.34  [6.01 – 39.13] | 1.65  [0.76 – 3.57] | 2.90  [0.84 – 10.01] | 3.76  [1.96 – 7.23] |
| Most of the time | 3.66  [2.07 – 6.48] | 4.34  [2.84 – 6.64] | 3.35  [2.15 – 5.20] | 3.73  [2.06 – 6.78] | 6.87  [3.44 – 13.72] | 4.22  [2.40 – 7.43] | 5.78  [2.24 – 14.93] | 2.78  [1.89 – 4.09] |
| Sometimes/  Almost Never | 2.04  [1.18 – 3.53] | 2.04  [1.24 – 3.34] | 2.78  [1.93 – 4.02] | 2.77  [1.66 – 4.61] | 3.32  [1.89 – 5.84] | 2.02  [1.25 – 3.26] | 2.96  [1.68 – 5.22] | 1.55  [1.04 – 2.33] |

^a^ Weighted to be nationally representative within each racial/ethnic group.

^b^ OR=Odds Ratio (unadjusted).

^c^ AI/AN=American Indian/Alaska Native; Black/AA=Black/African American; Hispanic/Latino ELP=Hispanic/Latino English

Language Preference; Hispanic/Latino SLP=Hispanic/Latino Spanish Language Preference; NH/PI=Native

Hawaiian/Pacific Islander.

Table S4. Racial/ethnic differences in associations between trust in the U.S. healthcare system and willingness to vaccinate against COVID-19 using interaction terms

|  | Always | Most of the time | Sometimes/Almost never |
| --- | --- | --- | --- |
|  | AOR [95% CIs]  p-value | | |
| Interaction terms |  |  |  |
| Trust*AI/AN (versus White) | 1.80 [0.57 – 5.71]  0.32 | 1.84 [0.72 – 4.75]  0.21 | 1.32 [0.53 – 3.27]  0.55 |
| Trust *Asian (versus White) | 0.60 [0.18 – 1.93]  0.39 | 0.58 [0.23 – 1.47]  0.25 | 0.38 [0.15 – 0.94]  0.04 |
| Trust*Black/AA (versus White) | 0.94 [0.37 – 2.43]  0.90 | 1.13 [0.54 – 2.39]  0.74 | 1.68 [0.82 – 3.43]  0.15 |
| Trust*H/L ELP (versus White) | 1.26 [0.33 – 4.84]  0.74 | 0.79 [0.30 – 2.06]  0.63 | 0.83 [0.33 – 2.08]  0.69 |
| Trust*H/L SLP (versus White) | 0.44 [0.12 – 1.69]  0.83 | 0.26 [0.08 – 0.84]  0.02 | 0.39 [0.12 – 1.22]  0.10 |
| Trust*Multiracial (versus White) | 0.41 [0.11 – 1.61]  0.20 | 0.97 [0.32 – 2.93]  0.96 | 0.65 [0.22 – 1.86]  0.42 |
| Trust*NH/PI (versus White) | 0.33 [0.10 – 1.05]  0.06 | 0.37 [0.15 – 0.95]  0.04 | 0.45 [0.19 – 1.07]  0.07 |

^a^ Weighted to be nationally representative within each racial/ethnic group.

^b^ AOR=Adjusted Odds Ratio.

^c^ AI/AN=American Indian/Alaska Native; Black/AA=Black/African American; H/L ELP=Hispanic/Latino English

Language Preference; H/L SLP=Hispanic/Latino Spanish Language Preference; NH/PI=Native

Hawaiian/Pacific Islander.

Table S5. Trust in the U.S. healthcare system and willingness to receive the COVID-19 vaccine across racial/ethnic groups (multigroup analysis)

|  | **Willingness to receive the COVID-19 vaccine** | | | | | | | |
| --- | --- | --- | --- | --- | --- | --- | --- | --- |
|  | **AI/AN** | **Asian** | **Black/AA** | **Hispanic/**  **Latino ELP** | **Hispanic/**  **Latino SLP** | **Multiracial** | **NH/PI** | **White** |
| **Trust in the U.S. healthcare system** |  |  |  |  |  |  |  |  |
| Always | 2.38  [1.14 – 4.96] | 3.00  [1.92 – 4.71] | 1.80  [1.09 – 2.97] | 4.71  [2.63 – 8.42] | 4.70  [3.00 – 7.38] | 2.56  [1.19 – 5.53] | 1.55  [0.73 – 3.28] | 3.45  [2.15 – 5.54] |
| Most of the time | 1.52  [0.97 – 2.37] | 3.19  [2.28 – 4.45] | 1.48  [1.07 – 2.04] | 1.94  [1.36 – 2.77] | 1.89  [1.31 – 2.73] | 2.50  [1.72 – 3.65] | 1.42  [0.92 – 2.19] | 2.02  [1.47 – 2.80] |
| Sometimes/  Almost Never | 0.64  [0.43 – 0.95] | 2.02  [1.37 – 2.98] | 0.93  [0.71 – 1.23] | 1.27  [0.90 – 1.81] | 1.53  [1.08 – 2.17] | 1.03  [0.72 – 1.49] | 0.83  [0.59 – 1.17] | 0.96  [0.68 – 1.36] |

^a^ Weighted to be nationally representative within each racial/ethnic group.

^b^ OR=Odds Ratio (unadjusted).

^c^ AI/AN=American Indian/Alaska Native; Black/AA=Black/African American; Hispanic/Latino ELP=Hispanic/Latino English Language Preference; Hispanic/Latino SLP=Hispanic/Latino Spanish Language Preference; NH/PI=Native Hawaiian/Pacific Islander.
